# A new technical approach for preparing frozen biological samples for electron microscopy

**DOI:** 10.1186/s13007-020-00586-5

**Published:** 2020-04-07

**Authors:** Othmar Buchner, Philip Steiner, Ancuela Andosch, Andreas Holzinger, Matthias Stegner, Gilbert Neuner, Ursula Lütz-Meindl

**Affiliations:** 1 Department of Biosciences, University of Salzburg, Hellbrunnerstrasse 34, 5020 Salzburg, Austria; 2 Department of Botany, University of Innsbruck, Sternwartestrasse 15, 6020 Innsbruck, Austria

**Keywords:** Cell organelles, High pressure freeze fixation, *Klebsormidium crenulatum*, *Lemna* sp., *Micrasterias denticulata*, *Pinus mugo*, *Ranunculus glacialis*, Transmission electron microscopy, Ultrastructure

## Abstract

**Background:**

Many methodological approaches have focused so far on physiological and molecular responses of plant tissues to freezing but only little knowledge is available on the consequences of extracellular ice-formation on cellular ultrastructure that underlies physiological reactions. In this context, the preservation of a defined frozen state during the entire fixation procedure is an essential prerequisite. However, current techniques are not able to fix frozen plant tissues for transmission electron microscopy (TEM) without interrupting the cold chain. Chemical fixation by glutaraldehyde and osmium tetroxide is not possible at sub-zero temperatures. Cryo-fixation methods, such as high pressure freeze fixation (HPF) representing the state-of-the-art technique for best structural preservation, are not equipped for freezing frozen samples. In order to overcome this obstacle, a novel technical approach for maintaining the cold chain of already frozen plant samples prior and during HPF is presented.

**Results:**

Different algae (*Micrasterias denticulata*, *Klebsormidium crenulatum*) and higher plant tissues (*Lemna* sp., *Ranunculus glacialis*, *Pinus mugo*) were successfully frozen and prepared for HPF at freezing temperatures (− 2 °C, − 5 °C, − 6 °C) within a newly developed automatic freezing unit (AFU), that we manufactured from a standard laboratory freezer. Preceding tests on photosynthetic electron transport and ability to plasmolyse show that the temperatures applied did not impair electron transport in PSII nor cell vitality. The transfer of the frozen specimen from the AFU into the HPF-device and subsequently cryo-fixation were performed without intermediate thawing. After cryo-substitution and further processing, the resulting TEM-micrographs showed excellent ultrastructure preservation of the different organisms when compared to specimens fixed at ambient temperature.

**Conclusions:**

The method presented allows preserving the ultrastructure of plant cells in the frozen state during cryo-fixation. The resulting high quality TEM-images represent an important step towards a better understanding of the consequences of extracellular ice formation on cellular ultrastructure. It has the potential to provide new insights into changes of organelle structure, identification of intracellular injuries during ice formation and may help to understand freezing and thawing processes in plant tissues. It may be combined with analytical TEM such as electron energy loss spectroscopy (EELS), X-ray analyses (EDX) and various other electron microscopic techniques.

## Background

The occurrence of freezing temperatures is one of the major environmental constraints limiting plant productivity and distribution [1]. A key point for survival of freezing temperatures is the tolerance of ice formation within the plant tissue that generally represents a dramatic incident. Extracellular ice, besides the mechanical impact, may expose plant cells to significant freeze dehydration [2]. Plants have evolved different strategies to cope with these freezing specific strain, such as freezing avoidance [3, 4] and freezing tolerance (reviewed by [5, 6]).

Since Levitt [7] there is a general agreement that ice, once it is formed intracellularly always causes cell death. In contrast, ice which is formed outside the cell wall (extracellularly) can principally be survived down to certain freezing temperature thresholds [1]. However, the causes of freezing injury to cells, the nature and cellular loci, are still unknown [8].

Extracellular ice causes a steep water potential gradient between the ice bulk outside the cells and the cell sap that consists of a supercooled highly diluted aqueous solution. The water potential of ice is much lower than that of unfrozen (supercooled) water at a specific freezing temperature and this water potential gradient becomes steeper with decreasing temperature [9, 10].

At slow cooling rates and due to the water potential gradient between ice and unfrozen water a certain amount of water will be withdrawn from the cell to the ice bulk resulting in cellular freeze dehydration [11] provided that the cell wall is not too rigid and allows cell volume reduction [12]. In the course of this, cells with thin and elastic cell walls can undergo massive but not necessarily lethal freezing cytorrhysis within a few seconds as was demonstrated in *Sphagnum capillifolium* leaflets [13].

While through the last decades many elucidating studies on freezing behaviour and freezing tolerance of plants have been performed (see [1, 6, 14–16]), only little is known on the consequences of extracellular ice formation on a cellular and sub-cellular level (e.g. [1, 17–19]). The effects of non-lethal extracellular ice-formation on plastids and mitochondria as well as on the endoplasmic reticulum and the Golgi-apparatus is widely unexplored.

For studying the cellular and subcellular responses to extracellularly freezing, and for gaining meaningful insights into mechanisms of freeze–thaw injury, the high resolution of TEM is required for the visualisation of cell membranes and cell organelles. As the current tools and techniques have been insufficient for preparing extracellularly frozen plant tissues for TEM, novel techniques have to be introduced.

As chemical fixation by glutaraldehyde (GA) and osmium tetroxide (OsO_4_) is not possible at freezing temperatures, a combination of cryofixation such as HPF or plunge freezing followed by cryo-substitution is the only possibility for preparation of frozen biological samples for TEM. The application of plunge freezing is limited to very small samples. Therefore, we have chosen HPF for the present investigation which is still state of the art in respect to structure preservation [20–28].

During HPF, cell suspensions or small tissue samples are exposed to high pressure (up to > 200 MPa) and almost simultaneously to rapidly (> 10 000 °Cˑs^−1^) cooling down to the temperature of liquid nitrogen (LN_2_, − 196 °C). This procedure causes the vitrification of the liquid water within and outside the cells. Despite outstanding issues [29], the principle of vitrification is also widely used in cryo-preservation and means that the water is directly transformed to a “glassy state” without forming ice crystals [30–33]. However, to our knowledge HPF was never applied to monitor frozen plant cells and tissues, and presently it is not fully clear what will happen to already formed ice (Ice Ih, hexagonal ice) when exposed to such high pressure and cooling rates. Principally, it is conceivable that under such conditions Ice Ih could be replaced by other types of ice (e.g. Ice II, Ice III) which have different crystal structures and physical properties, e.g. with regard to specific density [34, 35]. This could be relevant because transformations of the crystal structure of ice were shown to have the potential for disrupting cell structure [36].

The required technical approach for obtaining TEM-images of already frozen plant cells after HPF fixation is to keep frozen plant samples in a defined frozen state during preparation and fixation. Thawing of the samples during preparation and fixation must be strictly avoided. Hence, an important aim of this study was to develop a temperature controlled preparation technique including the insertion into the HPF-device. Such a tool is currently not available to our knowledge.

Our newly developed method was tested on four well known model organisms that have been widely studied by TEM before: *Micrasterias denticulata*, an unicellular freshwater alga (reviewed by [37]), *Klebsormidium crenulatum*, a filamentous aeroterrestrial green alga [38, 39], *Lemna* sp., a floating macrophyte [40–42], and the high alpine higher plant species *Ranunculus glacialis* [43, 44]. Additionally, needles from *Pinus mugo*, a coniferous shrub from the sub-alpine/alpine knee timber zone, were investigated.

We hypothesized that it should be possible to develop a novel technique to (1) keep frozen cells during the whole process of preparation and fixation by HPF in the frozen state. This must be considered as a prerequisite for analysis of the structural changes of cells, membranes and cell organelles while being exposed to extracellular ice. And further, after HPF fixation, without knowledge of the fate of extracellular ice during HPF, (2) we expected that these samples should provide good structural preservation of extracellularly frozen cells for TEM.

## Results

### Sample temperature

#### Temperature during the controlled freezing exposure prior to HPF

**Fig. 1:**
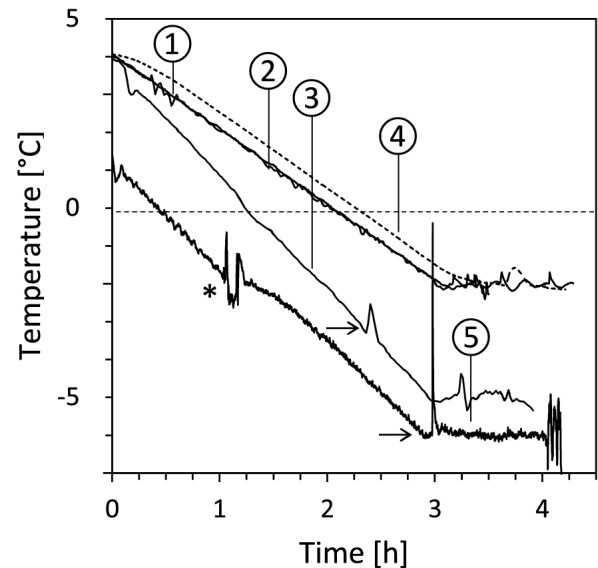
Sample temperatures during controlled freezing prior to HPF. Temperature of (1) *M. denticulata* and (2) *K. crenulatum* during linear cooling (− 2 °Cˑh^−1^) and subsequent preparation of the supercooled (unfrozen) algae at − 2 °C followed by inductance of ice formation (not shown). (3) Leaf temperature of *R. glacialis*. During linear cooling (− 3 °Cˑh^−1^) a clearly visible freezing exotherm, indicating extracellular freezing of the leaf mesophyll, occurred at -3.2 °C (horizontal arrow). (4) Temperature of *Lemna* sp. during linear cooling to − 2 °C (dotted). (5) Leaf temperature of *P. mugo* needles during freezing to − 6 °C (Arrow: freezing exotherm; asterisk: transient temperature deviations caused by technical reasons

During the experimental freezing exposure within an automatic freezing unit (AFU) that was newly developed by modifying a standard laboratory freezer, the leaf temperatures and the temperatures of the samples followed the preset-temperature course with high accuracy. The freezing experiments were started at temperatures between 0 °C and +4 °C. During the cooling phase down to the target freezing temperatures (*M. denticulata*, *K. crenulatum* and *Lemna* sp.: − 2 °C, *R. glacialis*: − 5 °C, *P. mugo*: − 6 °C) temperature deviations occurred with maximum ± 0.1 °C (*M. denticulata*, *K. crenulatum*), ± 0.4 °C (*Lemna* sp.) and ± 0.5 °C (*R. glacialis*). In case of *P. mugo* the maximum temperature deviations were temporarily higher (± 1.1 °C) due to technical reasons (temporary stabilisation at − 2 °C and working inside the AFU). Deviations of the sample temperature occurred also during sample preparation at target freezing temperatures, mainly when inserting and retracting the hands into/from the AFU. However, these deviations (typ. < ±1 °C) were only transient and did never lead to thawing of the samples (Fig. 1).

#### Temperature during transportation of the frozen samples to the HPF-device

**Fig. 2:**
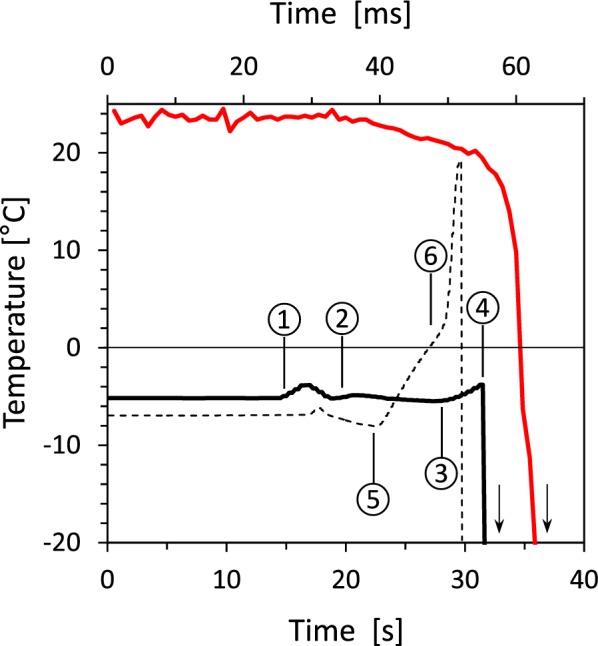
Typical temperature courses prior to and during high pressure freezing (HPF) of frozen samples. Data as generated during cryofixation by the HPF-device (red line, upper x-axes) and data based on direct measurements of the sample temperature using the temperature measurement adapter (TMA, black lines, lower x-axes), are compared. Solid black line: The frozen samples were transferred to the HPF-device within a precooled transfer block (0 to 15 s) to prevent thawing. When the loading device with the frozen sample was pulled out of the transfer block (1) short intermediate warming of the sample happened. Subsequently, the loading device was quickly positioned in the precooled loading area of the HPF-device and sample temperature declined again (2). During inserting the loading-device into the HPF-device (3) and locking it, sample temperature again slightly increased but remained well below 0 °C as before, because HPF was quickly released within only a few seconds (4). Dashed black line: Samples were treated as before, with the difference that the precooling of the interior of the HPF-device by the precooling device (PD) has been omitted. In this way the sample temperature rapidly began to rise immediately after inserting the loading device into the HPF-device (5) and the warming rate even increased after locking the specimen pod (6). Arrows indicate the further temperature courses (not shown) that ended at − 196 °C

A typical temperature course of a frozen sample (− 5 °C) during transportation from the AFU to the HPF-device and during HPF as measured by the temperature measurement adapter (TMA) is presented in Fig. 2.

Transport from the freezer to the HPF-device led to a slight but neglectable change of the sample temperature (− 0.2 °C). This was possible as transportation of the samples took place inside of a precooled metallic transfer block which was positioned inside the AFU during freezing and sample preparation. The transfer block effectively shielded the sample from the high ambient air temperature during transportation. By strictly following the protocol (see Table 1), also the next preparation steps, did not lead to significant temperature increases until HPF was started.

**Table 1:** Workflow for high pressure freezing of already frozen biological samples

Time	Action	Effects/notes
− 15 min	Set air temperature inside the Plexiglas^®^ chamber to 0 °C Insert a loading device and release HPF in advance	Slow precooling of the loading area
		Precooling of the internal components of the HPF-device
− 5 min	Insert and lock the precooling device (PD) Set air temperature inside the Plexiglas^®^ chamber to − 5 °C. The following disclosures relate to the experiment on *R. glacialis* and to the usage of the Leica Empact HPF-device (Leica Microsystems, Vienna, Austria)	Increased precooling of the loading area and of the internal components of the HPF-device
− 30 s	Fill LN_2_ directly into the pan (up to the rim)	Prevents the HPF-device from activating the release delay due to low LN_2_-level in the pan for 2–3 min
− 10 s	Switch the fan inside the Plexiglas^®^ chamber permanently on and remove the lid	Develops a cold (− 5 to − 10 °C) atmosphere around and above the loading area. Important for transferring the loading device from the transfer block to the loading area of the HPF-device
0 s	Unlock and remove the PD Pull out the loading device from the transfer block and quickly insert it into the HPF-device Promptly press ‘Lock’ and start HPF	This step must be executed within max. 8 s Otherwise the sample will thaw before HPF is completed

The following disclosures relate to the experiment on *R. glacialis* and to the usage of the Leica Empact HPF-device (Leica Microsystems, Vienna, Austria)

When the loading device with the sample was pulled out from the metallic transfer block and positioned on the moveable loading arm of the HPF-device, the sample temperature temporarily increased to − 3.8 °C and stabilized again at − 5.2 °C within 3 s. During inserting the loading device into the HPF-device, locking and starting HPF, the sample temperature again temporarily increased to − 4.2 °C before it fell down to − 196 °C during rapid fixation by HPF. In contrast, the temperature of a frozen sample increased rapidly already during sliding in by ca. + 10 °C and by further + 15 °C after locking the loading device, when precooling by the precooling device (PD) was not performed. It is worth noting that the temperature which is displayed and stored by the HPF-device may differ significantly from the real sample temperature as being measured by the TMA.

### Structural preservation of HPF frozen samples in TEM

By means of preceding vitality tests based on cell plasmolysis and on in vivo chlorophyll fluorescence combined with the visual assessment of freezing damage [45], the actual freezing resistance of the samples was determined. This was absolutely necessary for choosing temperature regimes (cooling rates, target temperatures, duration of exposure) to the samples that were not expected to cause visible frost damage to the plants examined.

**Fig. 3:**
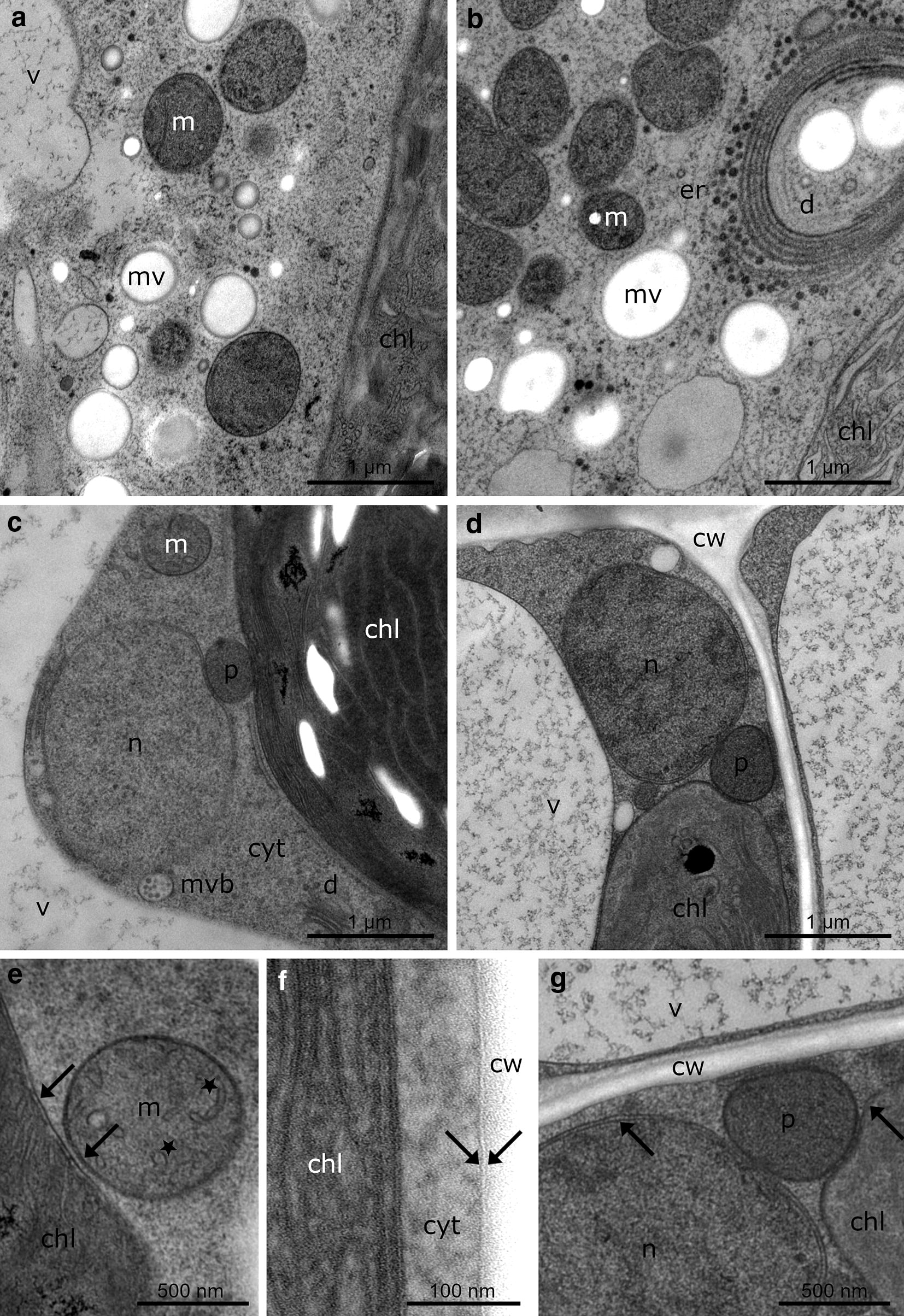
TEM micrographs of the alga *Micrasterias denticulata* (**a**, **b**) and the alga *Klebsormidium crenulatum* (**c**–**g**) after freezing (**b**, **d**, **g**) in comparison to 20 °C controls (**a**, **c**, **e**, **f**). **a** 20 °C control of *Micrasterias* shows single mitochondria (m), mucilage vesicles (mv), a part of the chloroplast (chl) and a part of the vacuole (v). **b***Micrasterias* cell after − 2 °C freezing with aggregated and fused mitochondria (m), endoplasmic reticulum (er), dictyosome (d), mucilage vesicles (mv) and chloroplast (chl). **c** 20 °C control of *Klebsormidium* shows vacuole (v) and cytoplasm (cyt) with single mitochondrion (m), chloroplast (chl), dictyosome (d), multi vesicular body (mvb), peroxisome (p) and nucleus (n). **d***Klebsormidium* after − 2 °C freezing shows chloroplast (chl), nucleus (n), peroxisome (p), vacuole (v) and cell wall (cw). **e** High magnification of 20 °C control of *Klebsormidium* shows double membranes (arrows) and cristae (asterisks) of mitochondrion (m) and chloroplast (chl). **f** High magnification of 20 °C control of *Klebsormidium* with chloroplast (chl) and cytoplasm (cyt), surrounded by the plasma membrane with the typical two-leaflet structure (arrows) and cell wall (cw). **g** High magnification of *Klebsormidium* frozen at − 2 °C shows peroxisome (p), vacuole (v), cell wall (cw) and double membranes (arrows) of chloroplast (chl) and nucleus (n)

**Fig. 4:**
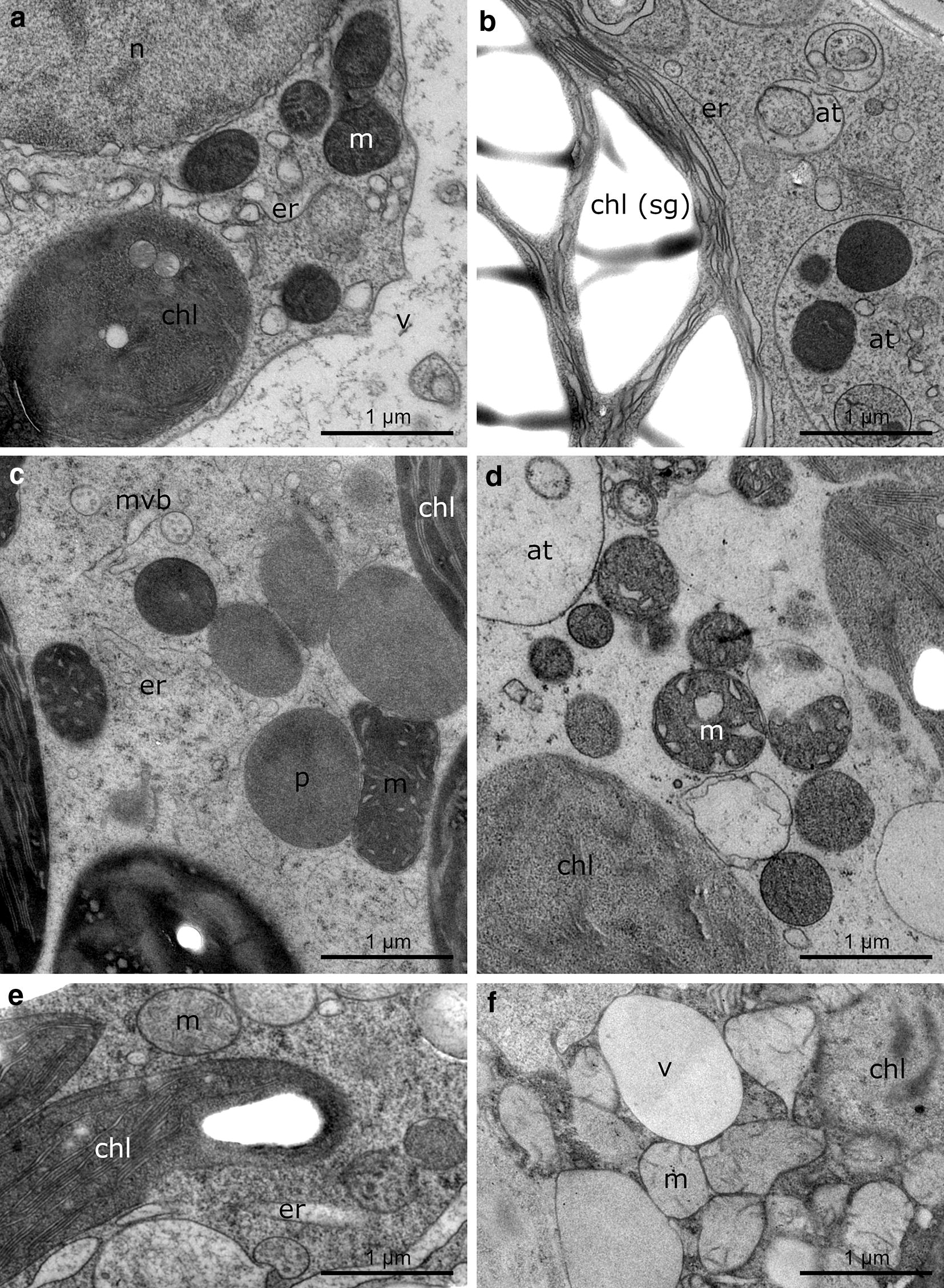
TEM micrographs of the higher freshwater plant *Lemna* sp. (**a**, **b**), the higher alpine plant *Ranunculus glacialis* (**c**, **d**) and the higher alpine plant *Pinus mugo* (**e**–**f**) after freezing (**b**, **d**, **f**) in comparison to temperate controls (**a**, **c**, **e**). **a** 20 °C control of *Lemna* shows vacuole (v), mitochondria (m), chloroplast (chl), endoplasmic reticulum (er) and nucleus (n). **b***Lemna* leaf after − 2 °C freezing shows chloroplast (chl) with enlarged starch grains (sg), endoplasmic reticulum (er) and signs of degradation and autophagic structures (at). **c** 4 °C control of *Ranunculus* shows mitochondria (m), peroxisome (p), multivesicular bodies (mvb), endoplasmic reticulum (er) and a part of the chloroplast (chl). **d***Ranunculus* leaf after − 5 °C freezing with aggregated mitochondria (m) and signs of degradation and autophagic structures (at). **e** 20 °C control of *Pinus* shows mitochondria (m), chloroplast (chl) and endoplasmic reticulum (er). **f***Pinus* needle after − 6 °C freezing shows chloroplast (chl), mitochondria (m) and vacuole (v)

During HPF of the unfrozen (control) and the frozen samples, pressurization and cooling speed were in the appropriate range (see Additional file 1). Both, in controls and in extracellularly frozen samples, structural preservation of the cytoplasm, cell organelles and cell walls corresponded to the high standard known from HPF fixed tissues and cells. Cytoplasmic structure and contents of organelles appeared electron dense and there were no indications of loss in material or structural damage (Figs. 3a–d, 4a–f) as postulated in earlier investigations [20, 22, 46, 47]. Particularly in *Micrasterias* (Fig. 3a, b) where HPF has been employed after exposure of the cells to different unfavourable environmental conditions and cellular inhibitors [42, 48–52], the results clearly show that the structural preservation of the extracellularly frozen cells is excellent and corresponds to that presented in the other studies. *Klebsormidium crenulatum* control cells (Fig. 3c, e-f) had a similar good preservation of the ultrastructure as -2 °C frozen samples (Fig. 3d, g). Also in *Lemna* (Fig. 4a, b) cytoplasmic and organelle structure are well preserved and correspond in quality to that obtained in a recent study on ionic stress effects [42]. For *K. crenulatum* (Fig. 3c, d) and *R. glacialis* (Fig. 4c, d) this is the first report on HPF fixed material as so far TEM-images are only available from chemically fixed samples (e.g. [53–55]). However, also in these plants all criteria demanded for excellent structure preservation seem to be fulfilled.

In the case of *P. mugo* (Fig. 4e, f) the situation is different. Whereas in the other objects used for the present investigation almost all frozen samples exhibited good structure preservation, the percentage of well-preserved tissues in *Pinus* was low, ranging in the area of 10%. This is rather due to problems with the infiltration of the needles by the resin than to insufficient fixation. In *P. mugo* particularly the duration of infiltration (see Materials and methods) as well as direction and velocity of sectioning determined the quality of the resulting sections and thus their suitability for TEM. When all criteria were met, structure preservation of both controls and extracellularly frozen *P. mugo* samples was sufficient (Fig. 4e, f). Comparison of the fixation quality with other studies is not possible, as to our knowledge all other TEM studies available on conifer needles were done by chemical fixation (see e.g. [56–59]). However, HPF fixed tissue of *Pinus contorta* during secondary wall formation or in the winter-dormant vascular cambium [60, 61], shows similar structure preservation as our controls and extracellularly frozen needles of *P. mugo*.

As expected, in the present study specific structural alterations could be observed in extracellularly frozen samples of all model plants which were not referable to the methodology applied but to the effects of low temperature and freezing (P. Steiner, U. Lütz-Meindl; personal communications). They mainly comprised changes in organelle structure and distribution as well as the appearance of different stages of autophagy and degeneration (Figs. 3a, b, 4b, d, f).

## Discussion

The present study clearly shows that our newly developed automatic freezing unit (AFU) makes it possible to preserve frozen plant samples for electron microscopy by HPF without interrupting the cold chain. The resulting TEM images provide excellent structure preservation. The study makes clear that both, transport of the sample from the AFU to the HPF-device and in particular the temperature inside its sample chamber are the main critical issues. Technical adaptations were particularly necessary in these cases to avoid an interruption of the cold chain of the samples during preparation.

During freezing and sample preparation inside the AFU the temperature stability was highly satisfactory and unintentional thawing was effectively prevented. However, inserting the hands into the AFU, in spite of wearing thermal insulating gloves always remained a challenging task for the temperature control mechanisms. The consequential temperature fluctuations could finally be minimized by slow and consecutively insertion and retraction of the hands. Whereas transportation of the samples within the metallic transfer block was unproblematic, the unavoidable mechanical contact of the loading device carrying the frozen sample, with the HPF-device required numerous tricks to avoid thawing of the sample. For cryo-fixation of frozen plant tissue it would be beneficial, if the next generations of HPF-devices could optionally be equipped with extended temperature controls for precooling the loading area and the relevant internal components.

It is well known that during cryo-fixation by HPF the cooling rate declines with the distance from the inner wall of the specimen carrier [46], which in turn may reduce the depth of vitrification and promote ice crystal growth. Furthermore, high pressure does not facilitate vitrification of water and diluted solutions [35] as being present for example in cell vacuoles and intercellular spaces of distinct plant tissues. As we abstained from using any cryoprotectant or other filling medium, we originally presumed that leaf tissue of *R.* *glacialis* and *P. mugo,* which is rich in air filled intercellular spaces, might show insufficient preservation quality (see [62]). This is not the case. One explanation for that could be that in *P. mugo* the leaf mesophyll cells are densely arranged and have robust cell walls. Principally, but to a lesser extent, this is also true for the palisade parenchyma layer of *R. glacialis*. Furthermore, in both cases it was possible to cut out tissue pieces which perfectly fitted into the gold plated specimen carrier (see Additional file 2) which may have minimized the thermal transfer resistance between its inner wall and the specimen. This however, poses a high challenge on the person who does the preparation. In case of *M. denticulata* and *K. crenulatum* the addition of a cryoprotectant was not necessary, because of the absence of air filled spaces around algae. This has been proven in numerous earlier studies. In summary, cryo-fixation of the frozen samples in case of *Micrasterias*, *Klebsormidium*, *Ranunculus* and *Lemna* was as successful as the fixation of the unfrozen controls. In this context attention shall be paid to the study of Yakovlev and Downing [63] who suggested that, during HPF, ice formation in the surrounding medium can enhance the cooling rate and therefore improve cryo-preservation. The more than threefold heat conductivity of ice compared to liquid water may thus have contributed to the good preservation of the structural integrity of the already (extracellularly) frozen samples.

Despite the well preserved cellular ultrastructure of the frozen samples, some questions concerning the fate of the hexagonal ice (Ice Ih), as being present in the frozen specimen before HPF takes place, remain open. The phase diagram of water is complex [64] and does not take into account dynamics like rapid changes of pressure and temperature. Therefore, a precise prediction on what will happen with Ice Ih during HPF cannot be given. It is known that water can supercool to − 92 °C at a pressure of 205 MPa and its melting point is decreased to − 22 °C [46, 65, 66]. And it is not fully clear whether the rapid pressurization during HPF induces amorphization of Ice Ih in a comparable way as described at 77 K and 1000 MPa by Mishima et al. [66]. At last it can’t be ruled out that ice in the frozen specimen became liquid during pressurization [67] for some milliseconds until final vitrification. As on the cellular level related responses can occur within very short time-spans [46], this option must not be completely discarded when evaluating the results.

On the other hand Bauer et al. [68] reported a density driven phase transition of Ice Ih to Ice II and Ice III at relatively high temperatures (170–230 K) when high pressurization rates up to 4000 MPaˑmin^−1^ were applied, which are lower than that observed during HPF (Additional file 1. 1: > 10^6^ MPaˑmin^−1^). They clearly proved a crucial impact of pressurization rate on the fate of normal hexagonal ice. Thus, during HPF a direct transition of already present Ice Ih to other ice forms or to a glassy state without intermediate melting appears possible.

Nevertheless, due to the lack of experimental studies concerning the behaviour of hexagonal ice during HPF conditions, presently this topic cannot be fully answered.

## Conclusions

We presented a method for adapting a commercial HPF-device for cryo-fixation of already frozen plant samples without intermediate thawing. The unicellular alga *M. denticulata*, the filamentous algae *K. crenulatum* and leaf tissues of the higher plants *Lemna* sp., *R. glacialis* and *P. mugo* were frozen by a software controlled, newly developed automatic freezing unit (AFU). The subsequent preparation for TEM took place within the AFU at different freezing temperatures and the following cryo-fixation by HPF was performed without intermediate thawing. The resulting electron micrographs show excellent preservation of all ultrastructural details and corresponded to those of unfrozen controls after cryo-fixation. The presented method is currently applicable to temperatures down to − 35 °C and has high potential for further studies on freezing effects on plants at an ultrastructural level. It may thus help to increase our knowledge on the different mechanisms causing freeze damage on a cellular level. Our results suggest that the method is also applicable to bacteria, fungi and animal tissue. Its use in combination with electron tomography (ET), focused ion beam-scanning electron microscopy (FIB-SEM) and analytical TEM such as energy-dispersive X-ray spectroscopy (EDX) and electron energy loss spectroscopy (EELS) may yield new insights into possible chemical changes of e.g. cell walls, metabolites or storage products during freezing.

## Methods

### Plant material

In order to demonstrate successful HPF of already frozen plant material we used five plant species on different evolutionary levels that inhabit contrasting ecosystems and are adapted to freezing temperatures to different degrees:

#### *Micrasterias denticulata* (Bréb.)

*Micrasterias denticulata*, an unicellular freshwater greenalga, inhabiting acid peat bogs up to an altitude of about 3000 m a.s.l. [69] has become a well-studied model organism in terms of cytomorphogenesis, cell physiology and stress response as well as ultrastructural research (for review see [37, 70, 71]). *M. denticulata* cells are large (200 µm in diameter) and reveal a highly ornamented, symmetric cell pattern that is manifested in two semi-cells and allows recognition of any environmental impact easily. Each semi-cell contains one large chloroplast and two large vacuoles. The cells were cultivated in a low concentrated liquid desmid nutrient solution [72] at + 20 °C and 100–150 µmol photons m^−2^·s^−1^ (time ratio day/night: 14/10 h).

#### *Klebsormidium crenulatum* (Kütz.) Lokhorst

*Klebsormidium crenulatum* (SAG 2415, Culture Collection of Algae, Göttingen, Germany) is an aeroterrestrial and desiccation tolerant, filamentous greenalga species which occurs in different terrestrial habitats (e.g. soil crusts). Samples were previously isolated from soil particles (Schönwieskopf near Obergurgl, Tyrol, 2.350 m a.s.l., 49° 50′ 59.88″ N, 11° 0′ 54.18″ E) and cultivated in modified Bold’s basal medium [73] at + 20 °C and a photosynthetic flux density (PPFD) 30 µmol photons m^−2^·s^−1^ (time ratio day/night: 16/8 h) until the experiments started.

#### *Lemna* sp.

*Lemna* is a widespread genus of free-floating aquatic freshwater plants and is frequently used for toxicological studies (e.g. [40, 42, 74]). *Lemna* was cultivated in Hoagland’s medium [75] at + 20 °C and PPFD 100–150 µmol photons m^−2^·s^−1^ (time ratio day/night: 12/12 h) until the experiments started. Leaves of *Lemna* were prepared for TEM.

#### *Ranunculus glacialis* L

*R. glacialis* is one of the highest ascending flowering plants (up to 4250 m a.s.l.) [76], and is well-studied in terms of its anatomy [53] and eco-physiology [44, 77–79]. It can be found in the upper alpine and nival zones of the European Alps but also in arctic and subarctic regions, where its fleshy and slightly succulent leaves are exposed to the risk of sporadic freezing during the entire vegetation period.

During summer whole individuals of *R. glacialis* were carefully excavated together with roots and surrounding soil from the summit area of the “Kleiner Isidor” (Stubaier Alps, Tyrol, 3150 m a.s.l., 46° 58′ 24.71″ N, 11° 06′ 27.88″ E). After transportation within a cooling box, the plants were put into a climate chamber and held at + 4 °C and a PPFD 80 µmol photons m^−2^·s^−1^ (time ratio day/night: 14/10 h) for 2 days until the experiments started.

#### *Pinus mugo* Turra ssp. mugo

*Pinus mugo* is a typical representative of the subalpine knee timber zone. It has a shrubby-like growth-habit and reaches a maximum height of ca. 3 m. During late winter, twigs (30–40 cm) were collected at Mt. Patscherkofel (Innsbruck, 1940 m a.s.l., 47° 12′ 38.4″ N/11° 27′ 5.98″ E), and transported to the laboratory within a snow-filled cooling box. Twigs were cut back under water and put into small plastic vials which were filled with tap water. Then each 3 of them were placed into 2 automatic freezing units (AFU) (see next chapter) which were precooled to + 4 °C until controlled freezing to target temperature was started.

### Sample exposure to freezing temperatures

**Fig. 5:**
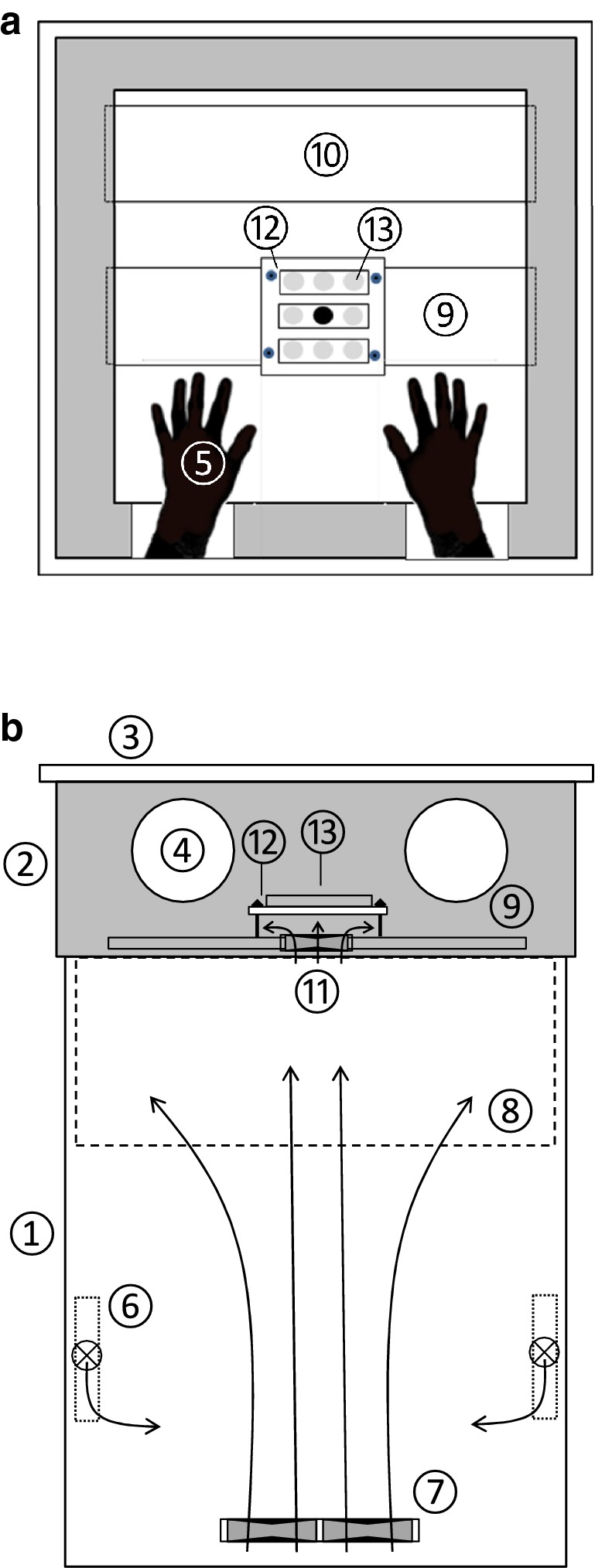
Automatic freezing unit (AFU) for controlled freezing and preparation of biological samples at freezing temperatures. **a** Top view. **b** Front view. The original lid of the laboratory freezer (1) was replaced by a top unit made out of thermally insulating material (2) which was covered by a detachable transparent Plexiglas^®^ pane (3). The top unit had two holes (4) through which thermally insulated gloves (5) for manipulation purposes were inserted. Temperature control was realized by two software controlled heating elements (6) which were equipped with miniature fans. Additionally, at the bottom of the freezer an array of four powerful fans (7) was placed to ensure good ventilation and temperature homogeneity within the freezer. A wire mesh (8) prevented the unintentional drop down of tools from the working (9) and the storage shelf (10). For freezing algal suspensions the working shelf was equipped with a fan (11) which blew air directly to the underside of the object carrier (12) to which three glass dishes with each three indentations (13) were placed. The central indentation, into which the control temperature sensor was plunged, was filled with NaCl-solution (black). For freezing of leaves a different working shelf without object carrier was used (not shown). Arrows indicate the main directions of the internal airflows

For controlled freezing of plants we developed an automatic freezing unit (AFU) from a common laboratory freezer (PLTA 0986, National Lab, Mölln, Germany; Fig. 5) which we equipped with two ventilated (HA40201V4-999, SUNON, Kaohsiung, Taiwan) heating elements (Nimbus B, 12 V/100 W, DBK David and Baader, Rülzheim, Germany). They were connected to a supply unit consisting of power supplies, commercial relays, modules for communications, control and temperature measurements (cRIO 9073: 266 MHz real time controller, NI 9264: 16 bit analog output modules, NI 9213: 16 channel thermocouple modules; all from National Instruments, Austin, TX, USA) and other necessary electronics. The temperature inside the freezer was continuously controlled and recorded by T-control software (Additional file 3), which we developed on a LabView platform (LabView 2012, National Instruments, Austin, TX, USA). Based on different algorithms the software allowed to precisely control (typically < ± 0.2 °C or lower) the temperatures inside the AFU by switching on/off the heating elements following the pattern of a PWM (pulse with modulation). In this way user defined temperature courses in terms of cooling and warming rates with a minimum target temperature − 35 °C at an ambient temperature + 25 °C could be realized. Furthermore, up to 32 fine wire thermocouple sensors (Type T, solder junction diameter < 0.2 mm, TT-Ti-40, Omega Engineering Inc., Stamford, USA) were connected to simultaneously record further relevant temperatures (e.g. leaf and alga suspension temperatures) by the system. For preparation of the samples at the selected freezing temperature, the AFU was permanently equipped with a highly thermal insulating (Styrodur^®^, BASF, Ludwigshafen, Germany) top unit covered by a detachable pane of transparent Plexiglas^®^ (20 mm, XT 29010, Röhm, Darmstadt, Germany) to optionally enable controlled illumination of the samples during freezing. The panelled side walls of the top unit had two adjacent horizontal holes (diameter: 120 mm). In these holes thermally insulating gloves (Thermo, HM Müllner, Eugendorf, Austria) were mounted. This allowed the preparation of frozen samples with minimized heat input into the freezing compartment and hence prevented undesired increases of the sample temperature. Inside the top unit a small bench and a storage shelf, both made of Plexiglas^®^, were located for preparation purposes and for keeping the preparation tools at a similar temperature as the samples.

In case of ***M. denticulata*** and ***K. crenulatum*** an additional pane (Plexiglas^®^) was mounted to the bench. On this pane, three small glass dishes (80 × 26 × 8 mm) with three pits each (diameter: 20 mm), for placing the algae, were positioned (Fig. 5). The central pit was filled with NaCl-solution. A fine-wire thermocouple sensor (see before) was plunged into it. This reference sensor was needed for control purposes only. It continuously measured the actual temperature of the NaCl-solution which was shown by preceding tests to be almost similar (± 0.2 °C) to the temperature of the adjacent alga suspensions. The NaCl-solution was required for surrounding the sensor by a liquid with a similar heat storage capacity, compared to the alga suspensions. This also prevents freezing, as the occurrence of a massive freezing exotherm at temperatures < 0 °C would have made it impossible to further control the suspension temperatures. Direct temperature measurement of the individual suspensions was not possible because the thermocouple sensors were shown to promote undesired ice nucleation and would have thus induced early and unpredictable freezing of the alga suspensions. The time of freezing and the desired ice formation temperature had to be controlled in any case (see next chapter).

We filled 1.5 ml of cell suspension (algae within their nutrient medium) into each (in total 8) indentation of the precooled (+ 4 °C) glass dishes and—after a stabilisation phase (e.g. 30 min)—cooled the samples down to target temperatures (*M. denticulata* and *K. crenulatum*: − 2 °C) which—as verified by preceding tests—were expected not to cause lethal damage to the cells. The cooling rate applied (− 2 °Cˑh^−1^; Fig. 1) is comparable with that used in other studies on algae (− 4 °Cˑh^−1^: [80], − 2.4 °Cˑh^−1^: [81]. When the target temperature was attained, preparation of the still unfrozen (supercooled) samples for HPF was started.

Whole individuals of ***Lemna*sp.** were put into small Eppendorf tubes which were filled with tap water. Temperature treatment started at + 4 °C and ended at − 2 °C (− 2 °Cˑh^−1^). Freezing was induced by dipping the tip of a cold dissection needle into the water to induce ice nucleation and freezing of the leaves.

For freezing of ***R. glacialis*** we placed the petioles of the leaves into small cups filled with tap water and a small amount of solution containing ice nucleating active (INA) bacteria (*Pseudomonas syringae*). This was done to prevent extended supercooling and to induce ice nucleation [82], and subsequently, freezing of the leaves within a temperature range corresponding well to the situation at the natural growing site (− 2 °C to − 3 °C; Stegner, Schäfernolte and Neuner, personal communication). Thermocouple sensors (see before) were mounted to the leaves by air permeable adhesive tape (Transpore™, 3 M, Österreich, Perchtoldsdorf, Austria). Then the samples were covered by a hemispherical glass bowl to reduce air movements and to promote the detection of freezing exotherms, indicating that extracellularly freezing of the leaves has taken place.

The minimum freezing temperature (target temperature) was specified based on the results of preceding tests for determining the actual freezing resistance of the sampled leaves following the protocol as described by Neuner and Buchner [45]. In vivo chlorophyll measurements (F_v_/F_m_) showed that the temperature threshold (LT_i_) at which initial freezing damage occurred, was at − 7 °C. We were aware that this kind of viability assessment would only provide information on the functionality of photosystem II (PS II). Temporary and reversible damage or impairment of cells or sub-cellular structures cannot be captured by this assay.

Thus, we exposed the samples to a temperature range starting at + 4 °C and ending at a target temperature of − 5 °C, which was higher by + 2 °C than LTi, at which initial freezing damage to PS II was expectable. The cooling rate applied (− 3 °Cˑh^−1^) (Fig. 1) is commonly used in stress physiological research on higher plants and close to that observed in nature [8, 82]. When the target temperature was attained, preparation of the already frozen leaf samples was started. Freezing of ***P. mugo*** in principle followed a similar procedure. The pre-determined LT_i_ was < − 20 °C, and so we applied the following temperature course: Start temperature: + 1 °C, target temperature: − 6 °C, cooling rate: − 3 °Cˑh^−1^.

### Sample preparation for high-pressure-freezing (HPF)

**Fig. 6:**
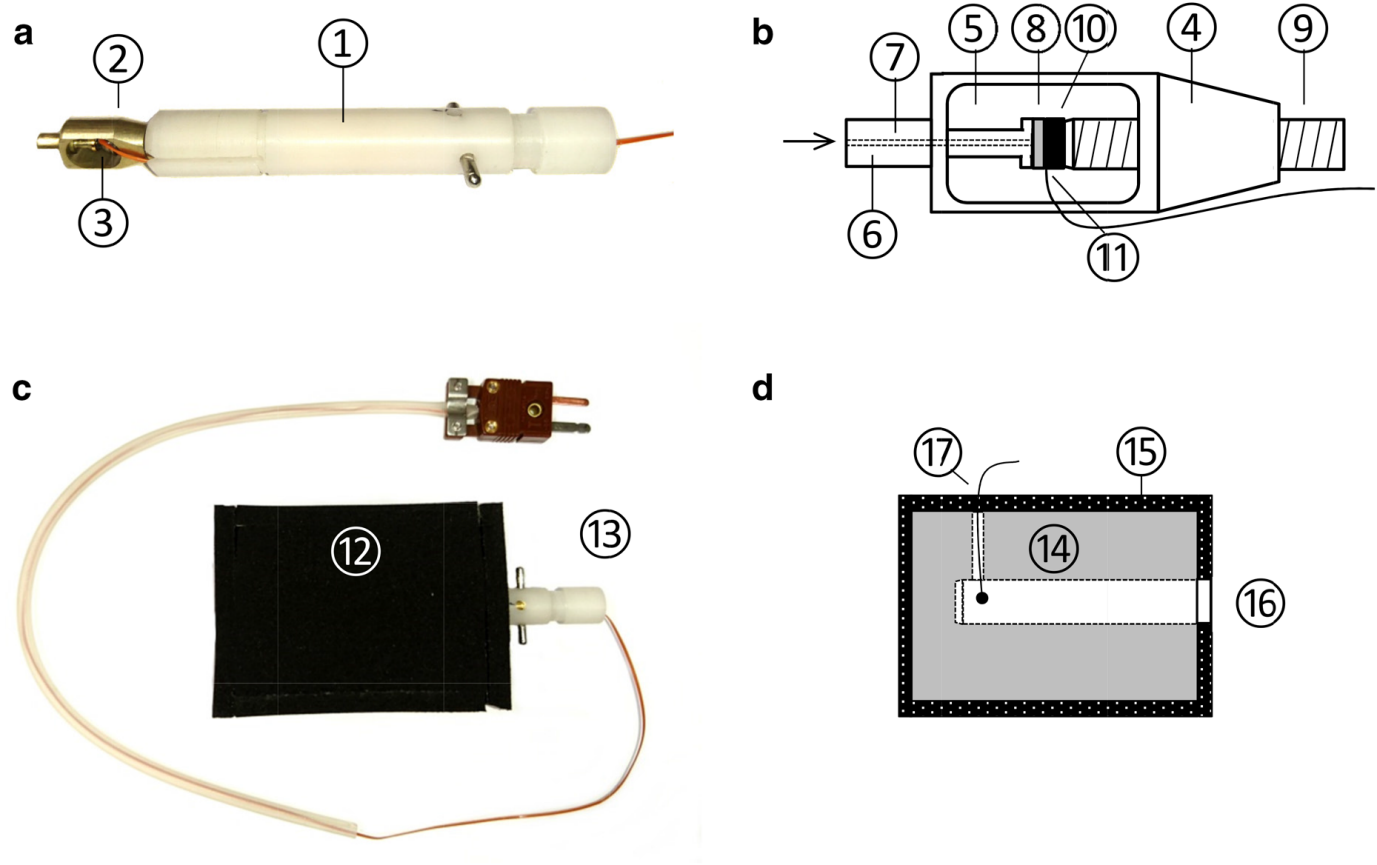
Adapter for measuring sample temperature during transfer within the transfer block and high pressure freezing. **a** Temperature measurement adapter (TMA) made from a standard loading device (1) consisting of a plastic tube with internal mechanical components and the specimen pod (2) with connected thermocouple sensor (3). **b** Specimen pod (schematic drawing, not to scale) (4) with two windows (5) through which jet streams of LN_2_ are applied and the pressure adapter (6) with an internal copper tube (7) for building up high hydraulic pressure (arrow) by methyl cyclohexane (liquid) which is directly forwarded to the bottom of the flat specimen carrier (8) which contains the sample. A grub screw (9) with an industrial diamond (10) on its tip seals the open side of the flat specimen carrier and ensures sufficient mechanical stability of the set-up. A thermocouple sensor (11) was connected to the flat specimen carrier to monitor sample temperature during transfer and high pressure freezing. **c** Transfer block (12) with affixed envelope of sponge rubber and inserted TMA (13). **d** Drawing (section) of the transfer block, showing the aluminium body (14), the sponge rubber envelope (15), the drill holes into which the loading device (16) and the thermocouple sensor for monitoring sample temperature during transportation (17) are inserted

The preparation of the samples took place within the AFU. In case of ***M. denticulata*** and ***K. crenulatum*** several cells or alga filaments, respectively, were collected from the still supercooled cell suspension and placed into a gold plated flat specimen carrier (interior dimensions: 1200 × 200 µm, item Nr. 16706897, Leica Microsystems, Vienna, Austria) of the high pressure freezer together with the surrounding nutrient solution. For transferring as many single cells of *M. denticulata* as possible, cells were wrapped in cotton fibres [47]. Ice nucleation was induced by carefully dipping the tip of a dissection needle into the alga suspension, thereby carrying over a tiny portion of ice crystals originating from the inner wall of the freezer. This did not lead to freezing of the algae cells themselves but only to freezing of the surrounding aqueous medium. The further preparation in principle followed the instructions of the manufacturer as described by Studer [83]. First, the flat specimen carrier with the frozen alga suspension was mounted to the specimen pod. Then the specimen pod was screwed to the loading device which was intermediately inserted into the hole of a precooled aluminium transfer block (Fig. 6) to keep the sample at the chosen freezing temperature during the impending transfer to the HPF-device (Leica Empact, Leica Microsystems, Vienna, Austria). The entire preparation procedure was conducted at − 2 °C, and was facilitated by a self-adapted binocular microscope (Stereo Star Zoom, Reichert, Vienna, Austria) which was placed on the transparent Plexiglas^®^ lid.

In case of *Lemna* sp., *R. glacialis* and *P. mugo* the procedure was similar. Small discs (diameter: 1 mm) of the leaves were punched out from the frozen leaf blade (*Lemna* sp., *R. glacialis*) by a special punching tool (item Nr. 706892, Leica Microsystems, Vienna, Austria). Then the frozen leaf discs were transferred into the gold plated flat specimen carriers (interior dimensions 1200 × 200 µm or 1200 × 400 µm, item Nr. 16706899, Leica Microsystems, Vienna, Austria) and processed in a similar manner as described for *Micrasterias* (see above). In case of *P. mugo* thin cross sections from the needles (ca. 3 cm from the tip) were cut using a standard razor blade, and each two of them were placed facing each other into a gold plated flat specimen carrier (interior dimensions 1200 × 400 µm, see above).

In all cases we did not add cryoprotectants such as sucrose and glycerol, because they are osmotically active and would have led to cell dehydration and undesirable consequences on the cellular ultrastructure [35, 46]. Recently, it was demonstrated by Yakovlev and Downing [63] that minimizing the amount of cryoprotectants may potentially improve preservation of cellular ultrastructure. Also the addition of the frequently used 1-hexadecene [84] was not performed because of its toxic effects on growing *Micrasterias* cells and the subsequent ultrastructural changes [47].

### High pressure freezing of the frozen samples

As it is not possible to directly measure and control the sample temperature during transfer from the AFU to the HPF-device and during the following process of high pressure freezing, the HPF-device had to be equipped with additional components. Extensive preceding tests were performed to develop a precisely defined workflow which ensures that thawing of the samples can be excluded.

#### Temperature measuring adapter for directly measuring sample temperature

As the cooling rate sensor of the HPF-device does not reliably reflect the actual sample temperature, we adapted a loading device for continuously measuring sample temperature directly at the flat specimen carrier. For that, we threaded a fine wire thermocouple sensor (see before) through the interior of the loading device and sandwiched the sensor tip to the flat specimen carrier. The resulting temperature measuring adapter (TMA, Fig. 6a, b) allowed us to monitor the sample temperature with high temporal resolution (250 ms), not only during the transfer from the AFU to the HPF-device but also during the whole HPF. This was necessary for developing the workflow (see below) which reliably ensures preservation of the sample temperature within a narrow range, even if it is not monitored.

#### Device for transferring the frozen samples at ambient temperature

Tests with the TMA showed that frozen samples (− 4 °C) mounted on a loading device thaw within < 3 s, when being exposed to ambient temperature (ca. + 25 °C). Therefore, to avoid thawing of the sample during transportation, we used an aluminium block (60 × 40 × 30 mm) which had a suitable drill hole (10 mm) into which a loading device could be inserted. Through a second drill hole (diameter 0.8 mm) a thermocouple sensor (see above) was inserted to measure internal air temperature of the 10 mm drill hole. The resulting transfer block was surrounded by self-adhesive sponge rubber (Fig. 6c, d) and located inside the AFU during freezing and preparation, so that its temperature was kept similar to the sample temperature. When preparation within the AFU was finished, the loading device was inserted into the transfer block and quickly moved to the HPF-device for further processing.

#### Cooling the loading area of the HPF-device

**Fig. 7:**
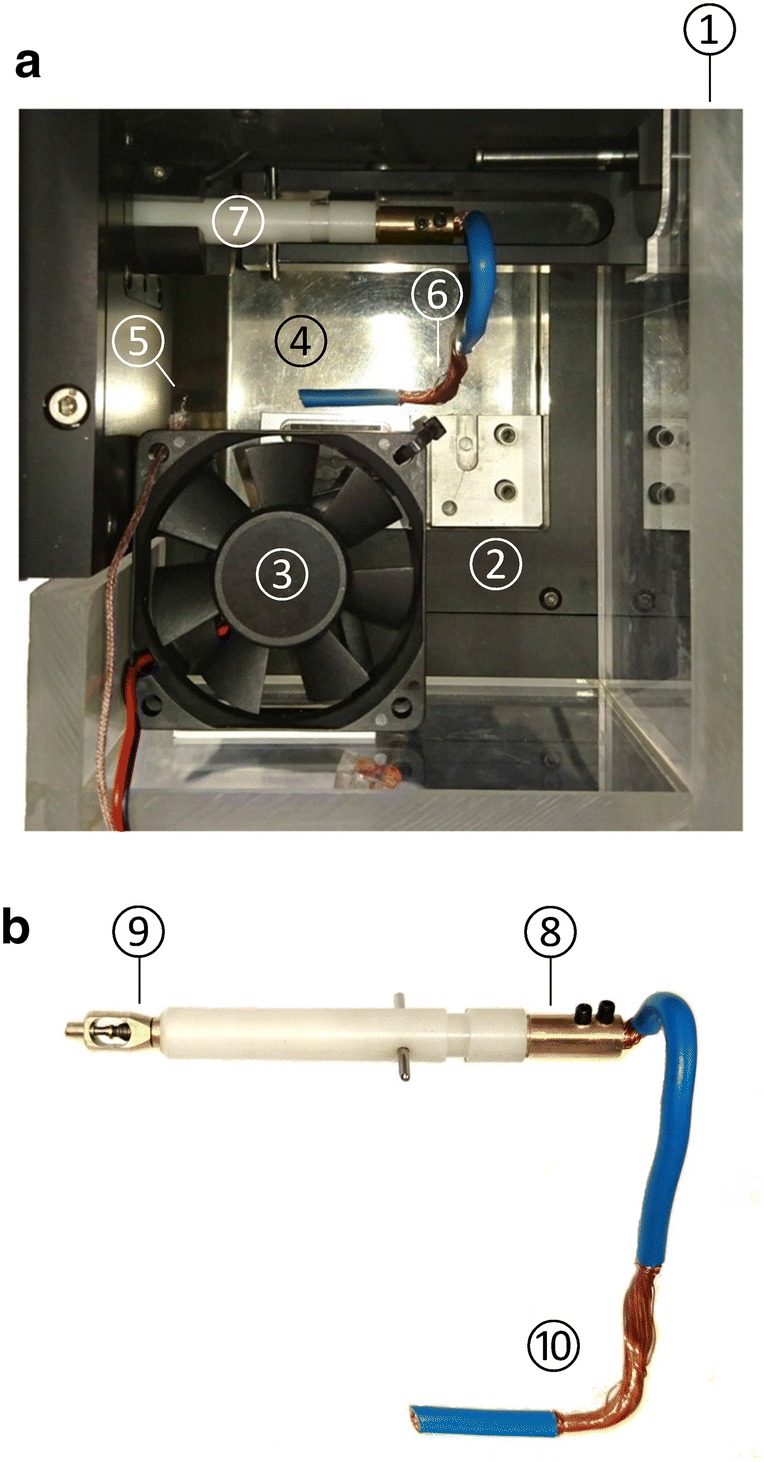
Components for cooling the HPF-device. **a** Cooling chamber for cooling the loading area of the HPF-device (top view, lid removed). Walls out of Plexiglas^®^ (1) enclose the entire loading area (2), while a fan (3) is generating airflow to the pan (4) which in normal operation is filled with LN_2_. As a consequence evaporation of LN_2_ will be increased and the loading area will rapidly be filled with cold, gaseous N_2_ which, on the other hand, cools all components and metal surfaces of the loading area, and prevents introduction of water vapour from the ambient atmosphere. For automatic temperature control a thermocouple sensor (5) is installed. The copper strains (6) of the inserted precooling device (PD) (7) lead to the pan and are surrounded by LN_2_. **b** PD made from a standard loading device. The internals were removed and replaced by an alloy rod (8) to which a standard specimen pod (9) and a copper strain (10) were screwed

Both static and moving parts of the whole loading area of the HPF-device needed to be precooled below the freezing point before a frozen sample on the loading device could be placed on the slider for insertion into the HPF-device. For this, a chamber (150 × 120 × 80 mm) of Plexiglas^®^ (10 mm) with a removable lid was built that enclosed the whole loading area of the HPF-device. The chamber was equipped with a thermocouple sensor (see above) and a small axial fan (KD1206PHS2, Sunon, Kaohsiung, Taiwan) which blew air vertically downwards to the pan filled with LN_2_ (Fig. 7a).

To control the air temperature inside the chamber, we used a simple on/off control provided by the T-control software: The fan was automatically turned on when the desired target temperature was exceeded. During operation, the fan rapidly increased the rate of evaporation of the LN2, creating a dense mist of cold, gaseous N_2_, that cooled all metal surfaces of the loading area until the temperature dropped below the desired temperature. Then the fan was automatically switched off until the desired target temperature was exceeded again.

Additionally, when removing the lid and keeping the fan operating continuously, also air temperature in close spatial vicinity to the opened chamber could be held well below the freezing point. In this way, the short-term transfer of the loading device from the transfer block into the precooled loading area of the HPF-device became possible without thawing of the sample.

#### Device for precooling the interior of the HPF-device

The last and most difficult step in realizing a continuous cold chain from sample preparation to HPF was cooling the internal components of the HPF-device which get in direct mechanical contact with the specimen pod after inserting and locking the loading device. The temperature of the components of the locking mechanism is normally between + 20 °C and + 30 °C due to the heat development inside the HPF-device by electronics and by special heating elements. To remove this heat and to effectively cool the related internal components without modifying the HPF-device, we designed a special precooling device (PD) which we inserted into the HPF-device 5 min before cryofixation started. The PD was built by modifying a normal loading device and replacing the internal components by a massive rod of alloy. To the tip of this rod a standard specimen pod was screwed in order to allow locking the PD, so that the locking mechanism could get into mechanical contact with it. The rear side of the alloy rod was made hollow, so that a copper strand (6 mm^2^) could be inserted and fixed by two transversal mounted screws (Fig. 7b). The other end of the copper strand was submersed in LN_2_. In this way, inserting and locking the PD resulted in a significant reduction of the internal temperature of the HPF-device and its locking mechanism. If subsequently the PD was quickly replaced by a loading device with a frozen sample, HPF of the sample was possible entirely at the required freezing target temperatures.

#### Workflow for HPF of frozen samples

Based on numerous preceding experiments using the TMA and PD a special workflow was established (Table 1). This workflow and in particular the related time frames therein must be strictly met to ensure that at any time intolerable temperature increase or even intermediate thawing of the sample will not occur.

## Sample preparation and TEM

After HPF, cryo-substitution took place in a LEICA EM AFS (Leica Microsystems, Vienna, Austria) freeze substitution device. Samples were submersed in cold acetone (− 80 °C) containing 2% osmium tetroxide and 0.05% uranyl acetate for 60 h. After this time the temperature was slowly (+ 10 °Cˑh^−1^) increased to − 30 °C, which was held constant for 4 h. Finally warming-up (+ 2.5 °Cˑh^−1^) was continued to + 20 °C. After washing with pure acetone and propylene oxide the samples were embedded into epoxy resin (medium grade; Agar Scientific, Essex, UK) and polymerized (+ 70 °C, 24 h). Then ultra-thin sections (70 nm) were prepared by an EM UC7 ultramicrotome (Leica Microsystems, Vienna, Austria) [47, 50, 85]. Micrographs were taken in a LEO 912 AB Omega transmission electron microscope (Zeiss, Oberkochen, Germany) equipped with an in-column energy filter and operated with a LaB_6_ cathode at 80 kV. Micrographs were filtered at zero energy loss. Images were captured by a CCD slow scan camera (TRS Sharpeye, Troendle, Moorenweis, Germany) and controlled by iTEM 5.0 software (Olympus SIS, Münster, Germany).

## Supplementary information

**Additional file 1: Figure S1.** Pressurization and cooling rate during high pressure freezing of already frozen samples of *M. denticulata* (− 2 °C) and *R. glacialis* (− 5 °C).

**Additional file 2: Figure S2.** Preparation of leaf sections from *Pinus mugo* for high pressure freezing.

**Additional file 3: Figure S3.** Virtual front-end of the T-control software.

## Data Availability

All data generated or analysed during this study are included in this published article and its additional files.

## Abbreviations

AFU: Automatic freezing unit
GA: Glutaraldehyde
EDX: Energy-dispersive X-ray spectroscopy
EELS: Electron energy loss spectroscopy
ET: Electron tomography
FIB-SEM: Focused ion beam-scanning electron microscopy
HPF: High pressure freezing
INA: Ice nucleating active
MCH: Methyl cyclohexane
PD: Precooling device
PWM: Pulse-with modulation
PPFD: Photosynthetic Photon Flux Density
TEM: Transmission electron microscopy
TMA: Temperature measurement adapter
