# Supplementary material for: A new technical approach for preparing frozen biological samples for electron microscopy
Source: Plant Methods. 2020 Apr 7;16:48. doi: 10.1186/s13007-020-00586-5 (PMC7137184; doi:10.1186/s13007-020-00586-5)

Fig. S1. Examples for pressurization and cooling rate during high pressure freezing of already frozen samples of *M. denticulata* (-2°C) and *R. glacialis* (-5°C).

Micrasterias denticulata

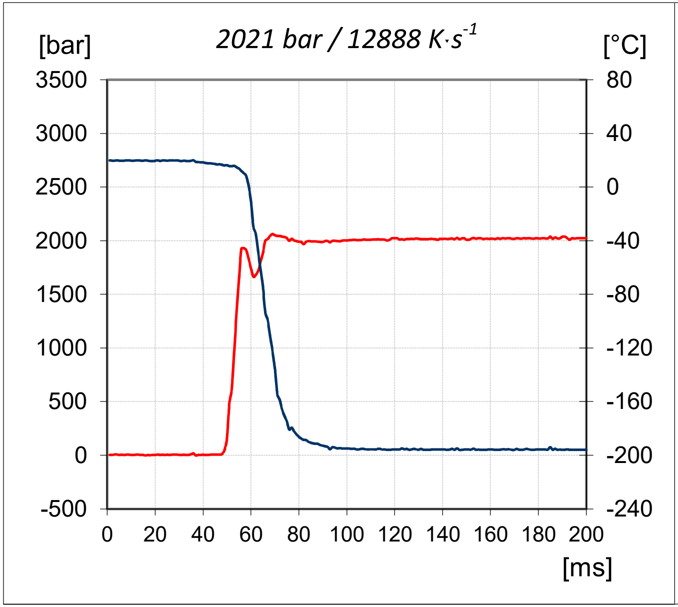

Ranunculus glacialis

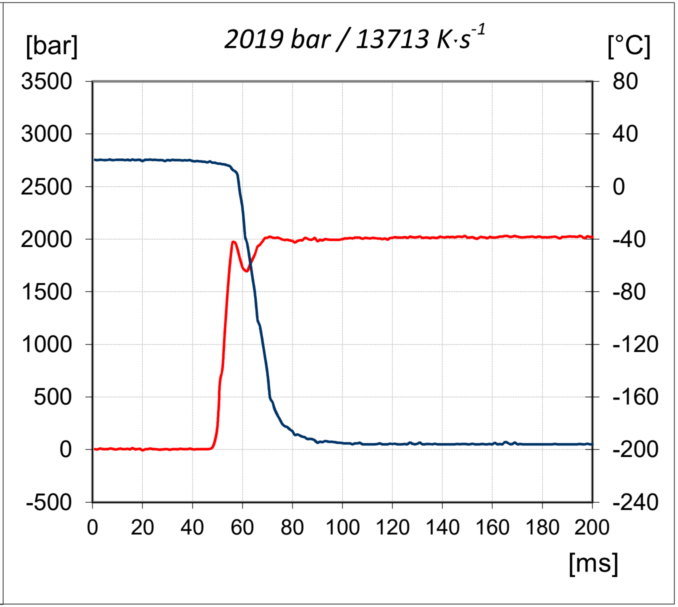

Supplement: Supplementary file 1 — Additional file 1: Figure S1. Pressurization and cooling rate during high pressure freezing of already frozen samples of M. denticulata (− 2 °C) and R. glacialis (− 5 °C). [file 13007_2020_586_MOESM1_ESM.pdf]
