# Supplementary material for: A new technical approach for preparing frozen biological samples for electron microscopy
Source: Plant Methods. 2020 Apr 7;16:48. doi: 10.1186/s13007-020-00586-5 (PMC7137184; doi:10.1186/s13007-020-00586-5)

Fig. S2. Preparation of leaves from *Pinus mugo* for high pressure freezing. Two equivalent cross sections were cut 3 cm from the tip of the leaves and symmetrically arranged in a standard gold plated flat specimen carrier (interior dimensions: 1200 x 400 µm) (1). To favour pressure build up and cooling rate, air cavities between the specimen (2) and the inner boundaries of the specimen carrier (3) were kept at a minimum.

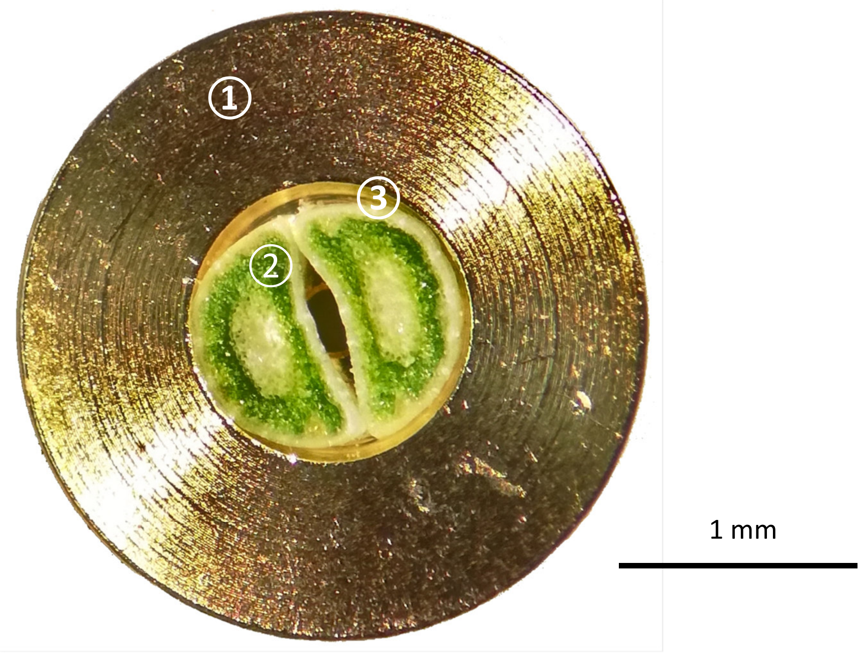

Supplement: Supplementary file 2 — Additional file 2: Figure S2. Preparation of leaf sections from Pinus mugo for high pressure freezing. [file 13007_2020_586_MOESM2_ESM.pdf]
