# Supplementary material for: A new technical approach for preparing frozen biological samples for electron microscopy
Source: Plant Methods. 2020 Apr 7;16:48. doi: 10.1186/s13007-020-00586-5 (PMC7137184; doi:10.1186/s13007-020-00586-5)
Supplement: Supplementary file 3 — Additional file 3: Figure S3. Virtual front-end of the T-control software. [file 13007_2020_586_MOESM3_ESM.pdf]

Fig. S3. T-control software. The virtual front-end of the software contains all necessary display and control elements for monitoring temperatures and other parameters and for programming temperature courses. (1) Control panel for programming the temperature course, for controlling the internal fans, for choosing the control temperature sensor and the control mode (PID, switch on/off) and for reading the set and the actual temperatures. Diagrams showing the programmed temperatur course (2), the actual temperature course (3), the actual control deviations (4) during the last 50 s and the actual temperatures of selected thermocouple sensors (5). A freezing exotherm alerter (6) produces an acoustic alarm if one of the selected temperature sensors records a freezing exotherm indicating the beginning of freezing of the related sample. If required the freezer and the heating elements can also be controlled manually (7) and it is also possible to add an offset temperature to the actual set temperatures (8). (9) Panel for controlling the temperature of the loading area of the HPF-device, (10) entry field for setting the control rate and the logging interval. (11) Panel for control of the automatic E-Mail alert system which sends a push E-Mail if control deviation surpasses a defined threshold. (12) Panel for controlling external illumination.

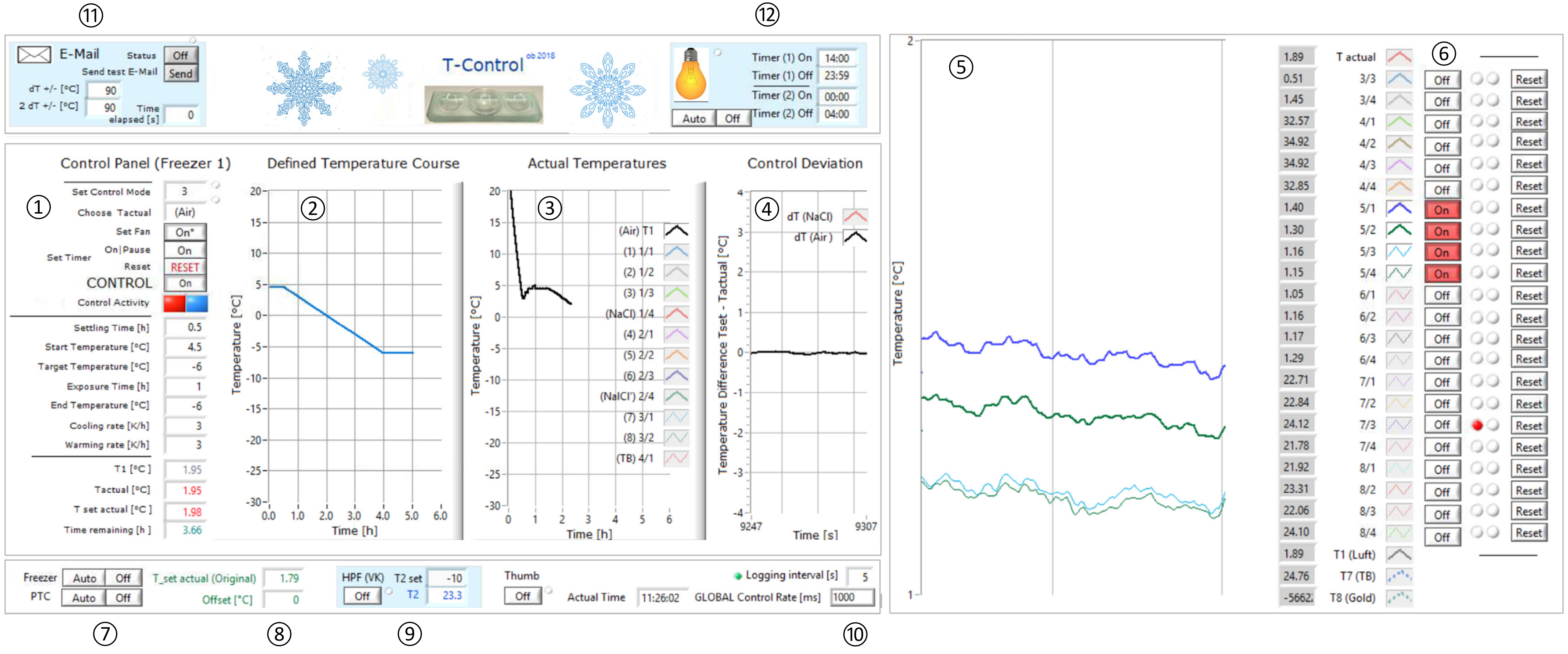

The T-control software was designed using LabView 2012 (National Instruments, Austin, TX, USA) and additional images from pixabay.com (published under the pixabay license which allows free private and commercial use).
